# Clinical Insights and Brief Research Report on Mesh Erosion Into Bowel Following Hernia Repair: A Single-Centre Series of Eight Cases

**DOI:** 10.3389/jaws.2025.15053

**Published:** 2025-11-07

**Authors:** Vidit Dholakia, Suvendu Sekhar Jena, Amitabh Yadav, Samiran Nundy

**Affiliations:** Institute of Surgical Gastroenterology, GI & HPB Onco-Surgery and Liver Transplantation, Sir Ganga Ram Hospital, New Delhi, India

**Keywords:** mesh complications, erosion, hernia surgery, fistula, abdominal wall hernias

## Abstract

**Background:**

Mesh erosion into the bowel is a rare but severe complication following hernia repair. Though synthetic mesh reduces recurrence rates, it carries risks of chronic infection, adhesion, and erosion. Literature is limited to isolated reports, and this case series aims to provide clinical insights into diagnosis and management challenges.

**Methods:**

We retrospectively reviewed eight patients with mesh-bowel erosion at a tertiary care centre in Delhi, India (2016–2025). Data on clinical presentation, surgical history, imaging, and management were analyzed. All patients underwent exploratory laparotomy with bowel resection and mesh removal when feasible.

**Results:**

The series included eight patients with a median age of 67 (range: 50–75). The time from initial surgery to erosion was highly variable, ranging from weeks to over 20 years. These complications arose from meshes placed in various anatomical planes, including onlay, preperitoneal, retrorectus, and intraperitoneal positions. The predominant clinical presentation was an enterocutaneous fistula (7/8 patients), with the small bowel as the most common erosion site. Preoperative imaging often underestimated the extent of erosion, which was confirmed intraoperatively. All patients required laparotomy; management included bowel resection (n = 7), mesh explantation (n = 6), and stoma formation (n = 5). One patient died from sepsis.

**Conclusion:**

Mesh erosion into the bowel, though infrequent, leads to significant morbidity and requires a high index of suspicion, especially in patients with vague abdominal complaints and history of hernioplasty. Timely diagnosis, aggressive surgical management, and multidisciplinary care are key to optimizing outcomes.

## Introduction

Mesh erosion into the bowel represents a rare yet significant complication that can arise after hernia repair surgery. The use of synthetic mesh has significantly reduced hernia recurrence rates from 50% to 10%–20% [1]. However, it has also introduced a spectrum of potential complications, including infection, adhesion, and erosion into adjacent organs [2]. Mesh erosion, characterized by the partial or complete penetration of the mesh into the bowel lumen. This condition may lead to significant morbidity, presenting with symptoms such as bowel obstruction, fistula formation, and chronic pain [3]. It is a rare but serious complication following hernia repair, and most existing literature consists of isolated case reports. There is a lack of consolidated clinical insight into the spectrum of presentations, the temporal variability of erosion, the intraoperative findings and challenges, the surgical decision-making required in such complex reoperations. This case series was conducted to fill the knowledge gap by presenting a comprehensive clinical and surgical perspective on mesh erosion from a tertiary care experience.

## Methods

We retrospectively reviewed the records of eight patients who presented with mesh erosion into the bowel between 2016 and 2025 at a tertiary Gastro-intestinal surgery centre in Delhi, India. No diagnosed cases from this period were excluded from the series. Data were extracted on demographics, prior surgical history, mesh type and placement, clinical presentation, imaging findings, intraoperative observations, and postoperative outcomes. Mesh erosion was defined as either intraoperatively visible penetration of mesh into bowel or histological evidence of mesh-induced bowel injury. All patients underwent exploratory laparotomy with resection of affected bowel segments and, where feasible, removal of the mesh. Ethics approval was waived as this was a retrospective, anonymized case series. Data regarding the material of the synthetic mesh and the precise technique of the original hernia repair were recorded when available. However, this information was frequently absent from patient records, reflecting a common challenge in retrospective analyses of complications from remote surgeries.

## Results

### Case 1

History & Presentation: A 63-year-old female with hypertension, prior incisional hernia repairs (1986, 1987), and gastroesophageal junction adenocarcinoma post-Ivor Lewis procedure (2008), at outside hospital, presented with abdominal pain, fever, and a discharging umbilical sinus. Specific records detailing the mesh type and indication for the original 1986 and 1987 repairs were not available. She had undergone multiple chemoradiotherapy cycles, with a metastatic recurrence in 2012 followed by an enterocutaneous fistula.

Evaluation: CECT showed two midline abdominal wall defects of 29 mm (EHS M2W1) and 52 mm (M4W2) with herniation of mesenteric fat and colon though the superior placed defect and a 46 × 40 × 42 mm collection. The previously placed mesh was not distinctly visualized amidst the dense inflammatory changes.

Surgery: In 2016, an exploratory laparotomy revealed a 4 × 3 cm lower abdominal wall defect with visible mesh and fecal discharge. A 10 cm segment of terminal ileum adherent to mesh was resected, and side-to-side anastomosis performed. Appendectomy was performed due to inflammation in the field and abdominoplasty with debridement of the chronic sinus tract was required to achieve a healthy soft tissue closure over the large defect.

Outcome: Postoperatively, she developed respiratory failure, renal dysfunction requiring dialysis, and sepsis. Despite intensive care, her condition deteriorated, and she was discharged against medical advice in a terminal condition with ongoing multi-organ failure. No further follow-up was possible, and her final outcome is unknown.

### Case 2

History & Presentation: A 60-year-old female with a history of total vaginal hysterectomy (1991), laparoscopic IPOM (2018)- for which the specific mesh type was not documented- and prior adhesiolysis with bowel resection for enterocutaneous fistula (ECF)- for which the exact year was unavailable in the records-all at outside hospital, presented with recurrent fistula. In January 2021, the patient experienced a recurrence of the enterocutaneous fistula.

**FIGURE 1:**
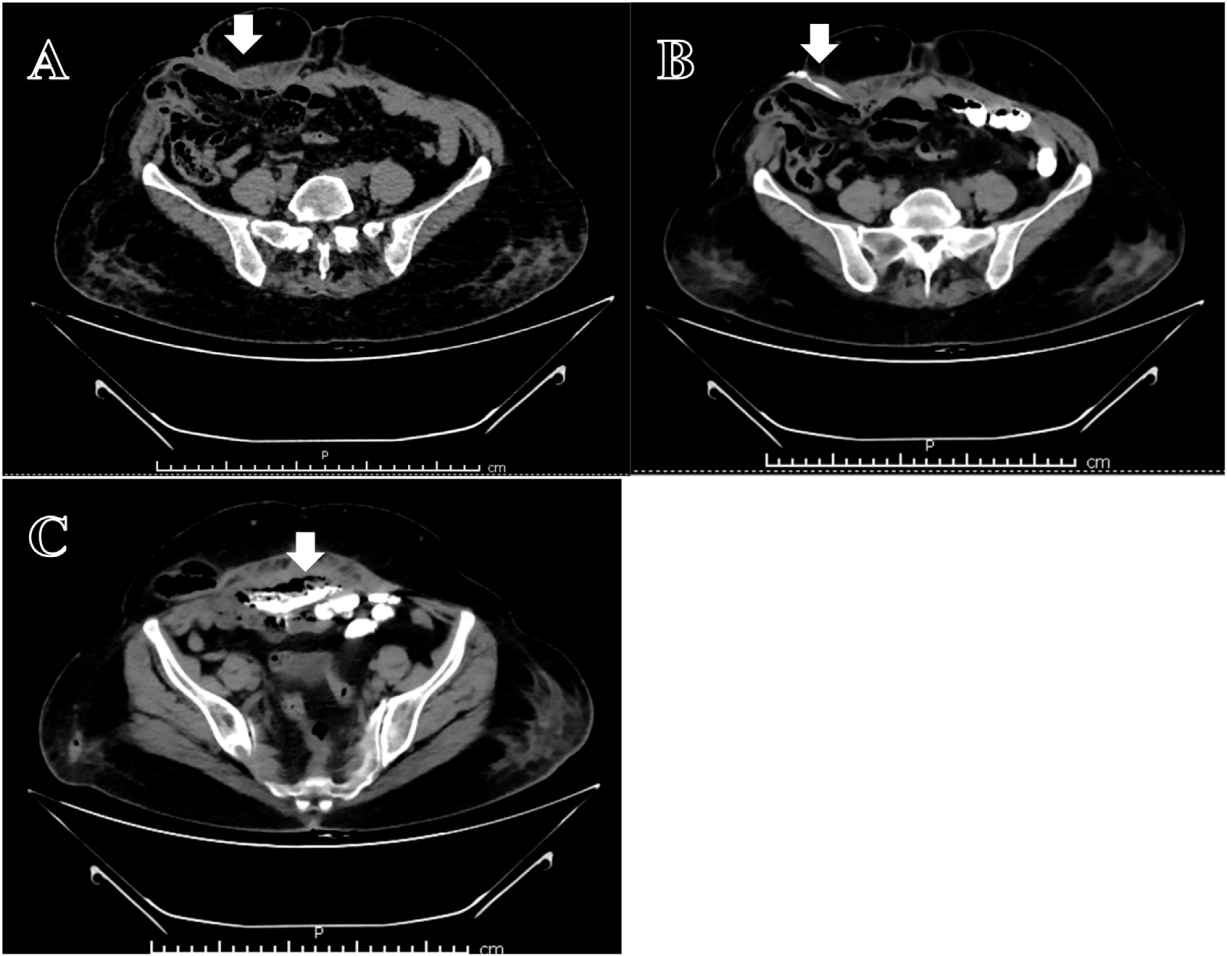
CT Fistulogram: **(A)** Collection below anterior abdominal wall with external communication (arrow). **(B)** Contrast fistulogram-showing fistula in right lateral abdominal wall (arrow). **(C)** Contrast filling in the air containing collection with communication with the bowel (arrow).

Evaluation: A CT fistulogram showed communication between the anterior abdominal wall, mesh, and distal jejunal loops [Figure 1].

Surgery: In 2021, she underwent an exploratory laparotomy. The procedure involved extensive adhesiolysis, complete mesh removal, and resection of the fistulous tract. This required the resection of multiple segments of densely adherent small bowel, totalling approximately 1.5 feet in length, which had eroded into the mesh. Following the resection, an ileo-ileal anastomosis was performed. A proximal double-barrel ileostomy was created from a segment of small bowel located approximately 3 feet from the duodenojejunal flexure.

Outcome: Postoperatively, the patient developed fever and pus discharge from the wound on POD 3, managed with broadspectrum antibiotics. On POD 9, stomal output increased leading to acute kidney injury (AKI) and hyperkalaemia on POD 18, which improved with nephrology input. Distal loopogram and subsequent feeds showed improvement, and the patient’s stomal output stabilized. Histopathological examination demonstrated serositis with a foreign body giant cell reaction surrounding the mesh, along with nonspecific inflammatory changes associated with the enterocutaneous fistula. The patient was discharged in stable condition, with follow-up care planned in the outpatient department. No further long-term follow up was available in the records.

### Case 3

History & Presentation: A 62-year-old female, with history of cholecystectomy (2004), appendectomy (2010), hysterectomy (2010) and with history of surgery for incisional hernia repair (2010) all at outside hospital; presented with abdominal pain and increased stool frequency for 7–8 months. Previous CT enterography (2021) revealed a probable ileo-colic fistula and liver irregularities.

Evaluation: Sigmoidoscopy suggested postoperative colonic stricture with possible colo-urethral fistula, confirmed by biopsy and Non-contrast Computed Tomography, showing soft tissue in the presacral region involving the bladder, ureters, and distal jejunal loops.

Surgery: She was admitted and after Double-J (DJ) stenting, she underwent exploratory laparotomy with adhesiolysis, take-down of a jejuno-colic fistula, mesh excision, primary repair of the jejunum, sigmoid resection, and ileostomy in July 2022. Operative findings included dense bowel adhesions and a fistula between the jejunum and sigmoid colon, where a previously placed mesh (2010-hernia surgery) had extruded. Biopsy revealed focal ulceration with serositis in the sigmoid colon and inflammatory granulation tissue around the mesh. Anatomical closure of abdominal wall was performed in midline.

Outcome: Postoperatively, a contrast leak was detected by NCCT on day 7, necessitating a second surgery with drainage of faecal collection, and identifying the leaks at the colo-colic anastomosis and the previous jejunal repair site. A double-barrel jejunostomy was created. After the second surgery, she was managed postoperatively with Ryle’s tube feeding, antifungal and antiviral therapy, and central line adjustments. After developing AKI with high stoma output and raised creatinine, ICU care and nephrology guidance led to improved renal function. She was discharged in a hemodynamically stable condition, tolerating oral feeds, with healthy stoma.

### Case 4

History & Presentation: A 66-year-old female with diabetes, hypertension, and prior Total abdominal Hysterectomy (TAH) (2005) underwent ventral hernioplasty (Intraperitoneal Dual layered mesh) with abdominoplasty in September 2023 for a long-standing incisional hernia (original EHS classification was not available in referral records). She developed small bowel obstruction 3 weeks after the surgery which was confirmed by Contrast enhanced CT, showing dilated loops with a transition at the mesh site. After adhesiolysis and serosal repair, she was discharged after further 2 weeks of admission (all at outside hospital). Operative records from this initial procedure detailing the extent of adhesiolysis were not available. The patient then presented at our centre, within 5 days with feculent wound discharge and obstruction symptoms.

Evaluation: Repeat imaging revealed a large anterior wall defect, mid-jejunum leak, and signs of chronic liver disease. Despite high-output fistula (∼2000 mL/day), she remained hemodynamically stable. Blood cultures grew *Acinetobacter* baumanii, and persistent fever warranted escalation of antibiotics.

Surgery: She underwent re-exploration, which revealed multiple small bowel fistulae, dense adhesions, and cirrhotic liver changes. About 2.5 feet of small bowel was resected; a double-barrel stoma and component separation were performed. Dense mesh incorporation prevented complete excision; negative pressure drains were placed near the residual mesh.

Outcome: She was managed in ICU, transitioned to ward, and discharged stable with distal stoma refeeding and follow-up planned.

### Case 5

History & Presentation: A 73-year-old diabetic, hypertensive female with prior TAH-BSO (Total abdominal Hysterectomy- Bilateral Salpingo-oophorectomy) (2001) and preperitoneal mesh hernioplasty (2012)- for which the original hernia type and specific mesh brand were not documented in the records-presented with weakness and reduced appetite for 3–4 months, followed by spontaneous rupture of a lower abdominal wall abscess on 31/08/2023. The initially serous discharge turned feculent.

Evaluation: MRI and CECT confirmed an infra-umbilical hernia with ECF from the mid-transverse colon.

Surgery: She underwent exploratory laparotomy with adhesiolysis, complete excision of infected mesh, transverse colon repair, ileostomy, and cholecystectomy (due to gallstones). Intraoperatively, a fistula between the transverse colon and anterior abdominal wall was found, with mesh densely adherent to colon and a large infected cavity. Abdominal wall closure was performed using Prolene sutures in interrupted manner.

Outcome: Postoperatively, she recovered well and was discharged stable. Ileostomy was closed at a later date without complications.

### Case 6

History & Presentation: A 50-year-old male with recurrent incisional hernias (repair with onlay mesh placement in 1999, 2007; EHS classification for the original hernias was not available in the records) following open appendectomy via McBurney incision (1990) presented with right lower quadrant pain and a bulge.

Evaluation: Contrast enhanced CT revealed caecal herniation and wall thickening (EHS L3W1). Colonoscopy showed an ulcerated caecal lesion and polyp.

Surgery: Exploratory laparotomy revealed dense adhesions and mesh erosion into the caecum with faecal fistula formation. Right hemicolectomy, mesh excision, and anatomical hernia repair were performed. Intraoperative frozen section ruled out malignancy.

Outcome: Postoperatively, he developed a loculated abdominal wall collection managed with IR-guided PCD on POD7. Symptoms of fever and oliguria resolved conservatively. Imaging on POD8 showed minimal residual collection without leaks. Final histopathology confirmed impacted mesh with no malignancy. He was discharged stable with drain *in situ* and follow-up advised.

### Case 7

History & Presentation: A 75-year-old male with prior laparoscopic inguinal hernioplasty (several years back)- for which the specific repair type and mesh used were not documented in the available records-presented in February, 2025 with weight loss, anorexia, and intermittent abdominal pain for 2 weeks.

Evaluation: Imaging (Positron Emission Tomography-CT, Ultrasound) showed a pelvic abscess with air foci, suggestive of a bowel fistula. Colonoscopy revealed a terminal rectal polyp.

Surgery: On 01/03/2025, laparoscopy revealed the cecal wall adhered to the right pelvic wall with a 3–4 mm fistulous opening and purulent collection anterior to the bladder. A ligaclip was noted in the mesentery, indicating prior surgical intervention. A lower midline incision revealed infected mesh bilaterally in the pubic region with ∼20 mL of pus. Mesh removal, appendectomy, and partial typhlectomy were performed.

Outcome: Postoperative course was uneventful; the patient resumed oral intake, remained afebrile and ambulatory, and was discharged with biovac drains for outpatient care. The drains were removed and no further long-term complications were noted in the available records at the time of this review.

### Case 8

History & Presentation: A 71-year-old female, with a history of hypertension, hypothyroidism, and two lower segment caesarean sections many years back, presented with a recurrent incisional hernia and discharging sinus at the infraumbilical region, along with intolerance to soft diet since last 1 week. She had undergone four prior incisional hernia repairs with mesh placement, the last was in 2013, all surgeries done at outside hospital. The exact years of the caesarean sections were not documented. For the four prior hernia repairs, specific details regarding the surgical techniques, mesh types, and EHS classifications were not available in the records.

Evaluation: CT imaging revealed intra-abdominal collections with air loculi, suggestive of an enterocutaneous fistula.

Surgery: She underwent exploratory laparotomy on 24/05/2025, which revealed extensive adhesions involving bowel loops, anterior abdominal wall, and liver. Two large hernial defects were noted in the lower midline and right lower abdomen. The mid-small bowel was densely adherent and showed multiple points of mesh erosion, resulting in a fistulous tract to the skin. The affected segment (∼1 foot) was resected and anastomosis was performed. The previously placed mesh was found in both retrorectus and intraperitoneal planes.

**FIGURE 2:**
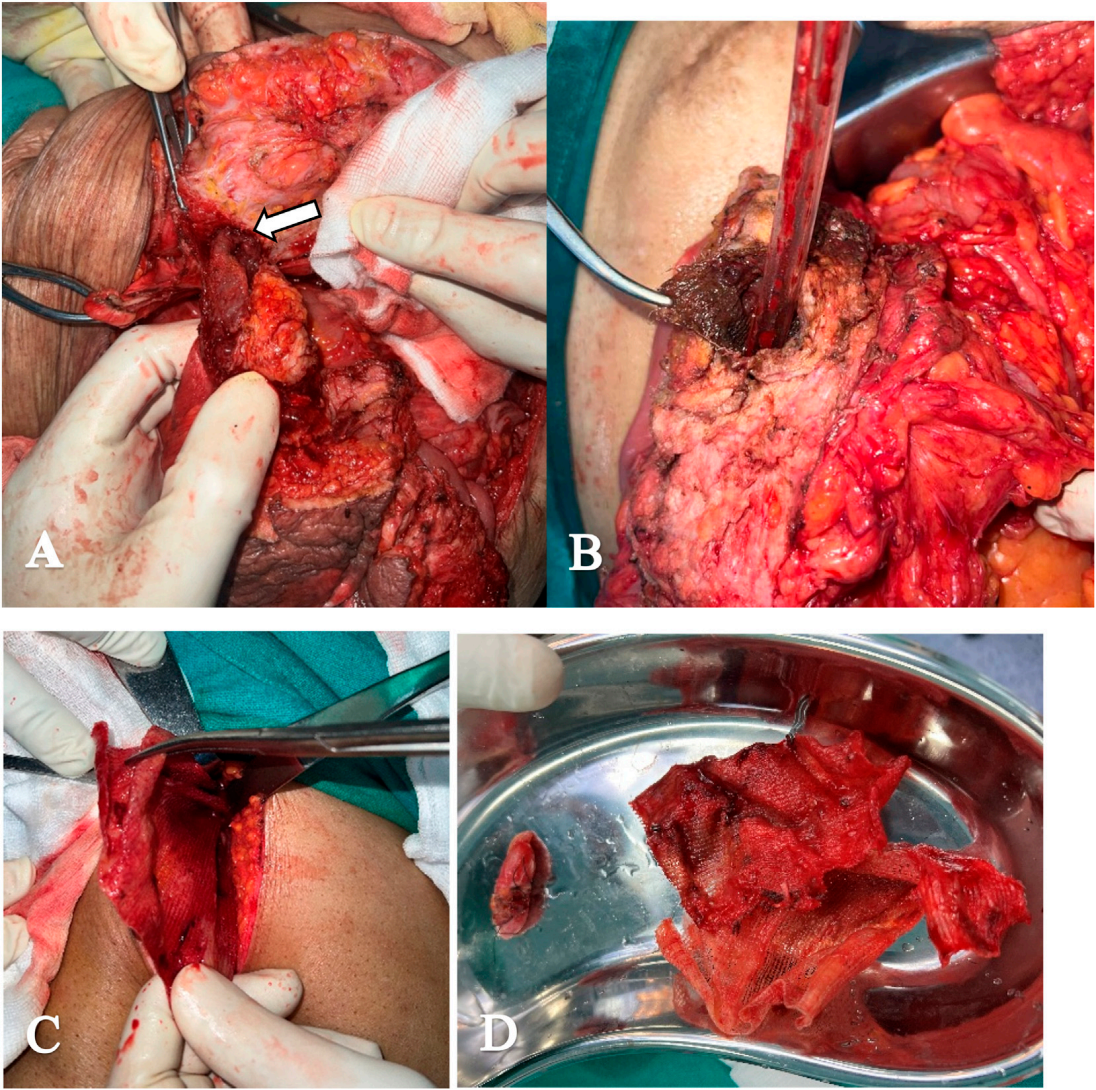
Intra operative findings. **(A)** Mesh incorporated into the bowel up-to the mucosal layer (arrow) (Case 8). **(B)** Mesh incorporated into cecum eroding the wall (Case 5). **(C)** After laparoscopic adhesiolysis of cecum and right colon, Retro pubic space entered-pus with infected mesh (Case 7). **(D)** Excised mesh along with part of cecum (Case 7).

Outcome: Postoperatively, the patient did well with a functioning stoma with good output and was discharged with no complications on further follow up [Figure 2].

### Summary of Outcomes

A summary of the key clinical features and outcomes for the eight-patient cohort reveals a wide spectrum of disease progression and complexity. The time from the initial mesh placement to the presentation of complications was highly variable, ranging from a few weeks to over two decades. The duration of symptoms prior to definitive surgical intervention also varied, from acute presentations over several days to chronic issues lasting for months. The predominant clinical finding was an enterocutaneous fistula, which was present in seven patients. These complications arose from meshes placed in various anatomical planes, including onlay (01), preperitoneal (03), retro rectus (01), and intraperitoneal (03) positions. The surgical management required was extensive, with 07 patients undergoing bowel resection, 06 having complete or partial mesh explantation, and 05 requiring the formation of an ostomy. Postoperative complications were common; 04 patients developed significant infectious complications, including sepsis and intra-abdominal collections. 01 patient required a re-operation for an anastomotic leak. The series had 01 mortality, with the patient succumbing to postoperative sepsis.

## Discussion

Abdominal wall hernia is best managed by reinforcement of wall using different materials of mesh, which can be placed in different anatomical layers of abdominal wall. It can be Onlay (overlying the defect), inlay (between the edges of the fascial defect) or underlay (underside the defect-which is pre peritoneal/intraperitoneal in case of abdominal wall). Recurrence rates associated with different placements are 44% in inlay, 23% in onlay group and12% in underlay group (12%) at (8–58) months [4]. Due to its lower recurrence rate, the underlay mesh placement is often preferred; however, this positioning also increases the risk of direct bowel contact and subsequent fistulisation.

Currently, more than 70 types of meshes are commercially available, constructed from synthetic materials (absorbable, non-absorbable, or a combination of both) and animal tissue. Despite reducing rates of recurrence, these meshes are associated with adverse effects like infection, adhesion, and bowel obstruction-fistula. Most meshes are chemically inert, nontoxic and non-immunogenic but none are biologically inert and as any foreign material would do, it triggers one or more of the following biological responses: (1) Destruction, (2) Inclusion, or (3) Rejection [4]. Foreign body reaction is a complex defensive response characterized by the involvement of foreign body giant cells, macrophages, fibroblasts, and capillaries, with their composition varying based on the shape and surface characteristics of the implanted material [5]. This interaction is characterized by three main aspects: Size of tissue reaction, cell density; and fibroblastic activity, which peaks one to 2 weeks post-wounding, usually on the 8th day for the intraperitoneal plane and on the 10th day for the extraperitoneal plane and achievement of optimum quantity of fibroblast needed for successful integration of mesh, takes about 2 weeks with further accumulation causing fibrosis associated with paraesthesia and pain. It leads to contraction and shrinkage of mesh, causing adhesions and fistula [6].

The occurrence of fistula formation can take a few months to several years with rates ranging from <1% in clean surgeries (<1–2%) up to 20% (severe contamination/infection) with rates mentioned in different literatures ranging 1%–6% [7]. The cases presented show a diverse array of clinical manifestations of mesh erosion, from abdominal pain, bowel obstruction, and fistula formation to more severe symptoms like chronic discharge and sepsis. The mesh can even migrate transmurally from abdominal wall into bowel [8] [Figure 1]. Primary mesh migration occurs due to inadequate fixation or external forces, with displacement risks heightened by early post-operative activities [9]. Fixation can reduce displacement but increases adhesion and nerve injury risks. Secondary migration results from foreign body reactions causing mesh erosion through tissue planes. Sharp mesh edges can weaken walls and erode viscera, triggering inflammatory responses [10]. Clinicians should consider mesh erosion or migration in patients presenting with unexplained regional pain or bowel/urinary symptoms following prior hernia repair.

In the cases mentioned, the initial symptoms were often nonspecific, complicating early diagnosis. The clinical presentations in our series were markedly varied, ranging from acute symptoms like bowel obstruction (Case 4) to more insidious signs such as a chronic discharging sinus (Cases 1, 8) or vague abdominal pain (Cases 3, 7). This heterogeneity underscores the diagnostic challenge in these conditions as low-grade mesh complications may initially mimic other gastrointestinal conditions. This emphasizes the importance of maintaining a high index of suspicion even with subtle presentations.

Mesh erosion and fistulisation often present with gas-filled fluid collections confirmed on imaging by oral contrast material extending outside the bowel lumen into fluid collections or abdominal wall tissues [11]. These cases underscore the diagnostic complexity of mesh erosion and fistula formation, which may not be fully appreciated through imaging alone.

CT imaging and fistulograms were crucial in identifying fistulous tracts and bowel involvement [Figure 1], but the extent of erosion was often only fully appreciated during exploratory surgery. For example, in Case 4, imaging revealed a large abdominal defect and adhered bowel loops, but the intraoperative findings of multiple small bowel fistulae and the inability to completely remove the mesh underscore the limitations of preoperative imaging in fully assessing the complexity of the situation. As seen in Case 6, the mesh erosion and incorporation into bowel wall may lead to diagnostic dilemma of malignancy which needs to be ruled out with biopsy and may require extensive surgical procedures. Similarly, in Case 7, colonoscopy and imaging showed only a small rectal polyp and pelvic collection, but surgery revealed a concealed caecal fistula and infected bilateral pubic mesh, confirming the underdiagnosed nature of low-grade erosive mesh complications.

Surgical intervention is the mainstay of treatment for mesh erosion into the bowel, with the complexity of the operation varying depending on the extent of erosion and associated complications [7]. The management requires removal of the infected mesh and addressing the intestinal fistula with resection and anastomosis or providing stoma [Figure 2]. The data to supporting mesh salvage using prolonged antibiotics and vacuum therapy or partial mesh excision are limited to case reports and case series and that too with short term follow up. Laparoscopic approach or hybrid approach can be feasible in some selective cases as in our case 7. The cases demonstrate a variety of surgical strategies, from bowel resection and anastomosis (Case 1, Case 2, Case 6, Case 7 and Case 8) to more extensive procedures like double-barrel stoma creation (Case 3 and Case 4) to manage recurrent fistulae and bowel compromise. Removal of infected or eroded mesh, as seen in Cases 2, 3, 5, and 7 is essential to reduce ongoing sepsis and further complications. In Case 7, partial typhlectomy and mesh removal were sufficient, and the patient had an uneventful recovery, which contrasts with the more severe postoperative courses of earlier cases and highlights the potential for better outcomes with early recognition and intervention.

The management of postoperative complications is equally important. For instance, postoperative infections, including wound infection and sepsis, as well as high fistula output, were common complications in these cases [4]. In Cases 5, despite the successful removal of the infected mesh and repair of the transverse colon, or as in Case 6, despite resection of pathological intestine with mesh, vigilant postoperative care was necessary to manage drain outputs and ensure the patient’s stable recovery. Case 7 also exemplifies this, where, despite underlying infection and mesh erosion, careful surgical handling and drain placement led to smooth recovery, reinforcing the value of thorough intraoperative debridement and effective postoperative care. Nutritional support, especially in patients with high-output fistulae or ileostomies, and the prevention of further infection are critical components of postoperative care.

Several risk factors for mesh erosion are identified in this series, including prior multiple abdominal surgeries, radiotherapy, and systemic conditions like diabetes and chronic liver disease. The increased adhesions and compromised wound healing in these patients may predispose them to erosion. Careful patient selection, choice of mesh material, and surgical technique can help reduce the incidence of this complication. When the mesh is placed intraperitoneal, the greater omentum, if not resected in previous surgery, can be placed in between the bowel and mesh as a protective barrier. Additionally, close postoperative monitoring for early signs of mesh complications is crucial for timely intervention. A significant challenge highlighted by our series is the frequent lack of data on the original mesh type and surgical technique. This missing information makes it difficult to ascertain whether factors like mesh material (e.g., polypropylene vs. ePTFE), pore size, or fixation method contributed to the subsequent erosion. Prospective, long-term registries of hernia repairs are needed to better identify these specific risk factors.

### Comparison With Existing Literature

When contextualized with recent literature, our series both confirms and expands upon the known spectrum of mesh erosion. The wide temporal range of erosion in our patients, from a few weeks to over 20 years, is notably broader than the 3- to 10-year timeframes reported in several case studies by authors such as Jallali et al. and Nair et al. [12, 13]. While their reports highlight the long-term, insidious nature of this complication, our series includes a case of acute erosion within weeks of surgery, underscoring that clinicians must remain vigilant for this complication in both the early and late postoperative periods. Furthermore, the clinical presentations in our series, dominated by severe septic complications like enterocutaneous fistulae and abscesses, contrast with some of the varied presentations reported elsewhere, which include chronic intermittent obstruction, anaemia from a mesh bezoar, and even asymptomatic findings discovered incidentally [12, 14]. This highlights the aggressive infectious potential of mesh-bowel erosion. Finally, the management strategies in our cohort align with a clear consensus in the literature. The consistent need for surgical intervention—primarily bowel resection and mesh explantation—in our patients echoes the approaches taken in every reported case, from enterotomy for mesh extraction to formal colectomy. This reinforces the principle that once mesh erodes into the bowel, aggressive surgical source control is almost always necessary to achieve a definitive resolution and prevent life-threatening morbidity.

A review of recent cases (2020 -2024) (Table 1) reinforces the variability in time to erosion (ranging from 3 to 10 years), with presentations including obstruction, anaemia, mesh extrusion, and asymptomatic findings. These reports consistently required surgical management—often bowel resection and mesh explantation—echoing the approach adopted in our series. Notably, mesh placed in intraperitoneal positions or under tension appears more prone to erosion. The variability in symptomatology and timing underlines the need for long-term monitoring and individualized decision-making in patients with prior mesh hernioplasty.

**TABLE 1:** Literature review: mesh erosion case reports.

Author/Year	Patient demographics	Type of mesh/Surgery	Time to erosion	Eroded into	Clinical presentation	Management
Chandrasinghe et al., 2020 [14]	71M	Composite mesh, ventral hernia repair	5 years	Proximal ileum	Asymptomatic, mesh found incidentally	Enterotomy with mesh extraction
Holder-Murray et al., 2022 [2]	65M	Composite mesh, parastomal hernia	3 years	Small bowel	Chronic intermittent obstruction	Enterotomy, mesh removal, partial colectomy
Nair et al., 2022 [12]	74M	Synthetic mesh, ventral hernia repair	10 years	Small bowel	Anemia, mesh bezoar, chronic symptoms	Resection of bowel and mesh, reconstruction
Zhang et al., 2023 [15]	67F	Keyhole mesh, parastomal hernia	3 years	Colon	Abdominal pain, mesh expelled *per rectum*	Resection of colon, colostomy revision
Jallali et al., 2024 [13]	52F	Polypropylene mesh, sublay repair	10 years	Small bowel	Obstruction, pain, mass	Bowel resection and mesh removal
Present Study	7 patients, M/F mix (50–75 years)	Various (IPOM, Onlay, open mesh)	Weeks to >15 years	Jejunum, ileum, colon, caecum	Fistula, abscess, obstruction, fever	7 laparotomies, 6 resections, 5 mesh explants

### Limitations

We acknowledge the limitations of our study. Its retrospective design and small sample size of eight cases are inherent constraints when studying a rare complication. Furthermore, as a tertiary referral centre, some patient data regarding initial surgeries were incomplete, and long-term follow-up was not uniformly available. Despite these limitations, this series provides valuable, in-depth insights into the diagnosis and complex management of this condition.

### Conclusion

Mesh erosion into the bowel, though rare, is a severe complication that significantly increases patient morbidity. Early recognition, multidisciplinary evaluation, prompt surgical intervention, and meticulous postoperative care are critical to improving outcomes. This series underscores the need for heightened clinical suspicion in patients presenting with vague gastrointestinal symptoms following hernia repairs, even in the absence of significant comorbidities. A multidisciplinary approach involving surgeons, radiologists, and critical care specialists is essential for successful outcomes in these patients.

## Data Availability

The original contributions presented in the study are included in the article/supplementary material, further inquiries can be directed to the corresponding author.

## References

[1] Gandhi D Marcin S Xin Z Asha B Kaswala D Zamir B . Chronic Abdominal Pain Secondary to Mesh Erosion into Cecum Following Incisional Hernia Repair: A Case Report and Literature Review. Ann Gastroenterol (2011) 24(4):321–4. 24713759 PMC3959323

[2] Lutfi W Griepentrog JE Russavage JM Salgado J Holder-Murray J . Complete Mesh Migration into the Small Bowel Following Parastomal Hernia Repair. ACS Case Rev Surg (2022) 3(6):86–90.

[3] Klein J . Encasement of Ventral Hernia Mesh Within Small Bowel: A Case Report. Int J Surg & Surg Tech (2024) 8(2):1–3. 10.23880/ijsst-16000223

[4] Schumpelick V Fitzgibbons RJ . Hernia Repair Sequelae. Berlin/Heidelberg, Germany: Springer (2010).

[5] Anderson JM . Biological Responses to Materials. Annu Rev Mater Res (2001) 31(1):81–110. 10.1146/annurev.matsci.31.1.81

[6] Zogbi L . The Use of Biomaterials to Treat Abdominal Hernias. In: Biomaterials Applications for Nanomedicine. London, United Kingdom: IntechOpen Limited (2011).

[7] Arnold MR Kao AM Otero J Marx JE Augenstein VA Sing RF Mesh Fistula After Ventral Hernia Repair: What Is the Optimal Management? Surgery (2020) 167(3):590–7. 10.1016/j.surg.2019.09.020 31883631

[8] Lee Y Bae B-N . Transmural Mesh Migration from the Abdominal Wall to the Rectum After Hernia Repair Using a Prolene Mesh: A Case Report. Ann Coloproctol (2021) 37(Suppl. 1):S28–33. 10.3393/ac.2020.04.19 32674553 PMC8359694

[9] Agrawal A Avill R . Mesh Migration Following Repair of Inguinal Hernia: A Case Report and Review of Literature. Hernia (2006) 10(1):79–82. 10.1007/s10029-005-0024-8 16258705

[10] Kurukahvecioglu O Ege B Yazicioglu O Tezel E Ersoy E . Polytetrafluoroethylene Prosthesis Migration into the Bladder After Laparoscopic Hernia Repair: A Case Report. Surg Laparosc Endosc Percutan Tech (2007) 17(5):474–6. 10.1097/SLE.0b013e3180f62f56 18049421

[11] Gavlin A Kierans AS Chen J Song C Guniganti P Mazzariol FS . Imaging and Treatment of Complications of Abdominal and Pelvic Mesh Repair. Radiographics (2020) 40(2):432–53. 10.1148/rg.2020190106 32125951

[12] Nair I Slater K . When Hernia Mesh Erodes into the Bowel: A “Bezoar” Case. Int J Abdom Wall Hernia Surg (2022) 5(3):150–3. 10.4103/ijawhs.ijawhs_88_21

[13] Jallali M Chaouch MA Zenati H Hassine HB Gafsi B Noomen F . Complications Unveiled: A Detailed Case Report on Mesh Migration Post-incisional Hernia Repair. Int J Surg Case Rep (2024) 121(109976):109976. 10.1016/j.ijscr.2024.109976 38954968 PMC11263626

[14] Chandrasinghe P De Silva A Welivita A Deen K . Complete Migration of a Composite Mesh into Small Bowel Incidentally Found During Laparotomy for Colectomy in an Asymptomatic Patient: A Case Report. J Med Case Rep (2020) 14(1):207. 10.1186/s13256-020-02540-4 33126917 PMC7602325

[15] Zhang Y Lin H Liu J-M Wang X Cui Y-F Lu Z-Y . Mesh Erosion into the Colon Following Repair of Parastomal Hernia: A Case Report. World J Gastrointest Surg (2023) 15(2):294–302. 10.4240/wjgs.v15.i2.294 36896303 PMC9988641

